# Predicting patellar kinematics and contact forces after TKA: a simulation study on quadriceps malalignment

**DOI:** 10.1186/s42836-026-00407-5

**Published:** 2026-07-07

**Authors:** Florian Michaud, Ánxela Pérez Costa, Daniel Dopico, Simon Talbot

**Affiliations:** 1 Laboratory of Mechanical Engineering, CITENI, Campus Industrial de Ferrol, Universidade da Coruña, Ferrol, 15403 Spain; 2 Department of Orthopaedic Surgery, Western Health, Footscray, VIC 3011 Australia

**Keywords:** Patella instability, Extensor mechanism, Contact forces, Arthroplasty, Total knee replacement, Patellar tracking, Computer simulation, Multibody dynamics

## Abstract

**Background:**

Patellofemoral complications remain a frequent cause of dissatisfaction following total knee arthroplasty (TKA), despite continuous advances in implant design and surgical techniques. Abnormal patellar tracking may lead to increased contact stresses, instability, and anterior knee pain, particularly in patients with preoperative quadriceps malalignment. Subject-specific assessment tools are therefore needed to improve surgical planning and postoperative outcomes.

**Methods:**

A subject-specific multibody dynamics (MBD) framework was developed to simulate patellofemoral mechanics following TKA. A single representative patient with pronounced quadriceps malalignment was selected for this proof-of-concept study to enable detailed subject-specific modeling and experimental validation under controlled conditions. The effects of femoral and tibial component positioning, including internal/external rotation and varus/valgus alignment, were systematically evaluated. Numerical predictions were validated using a sensorized 3D-printed knee rig.

**Results:**

Quadriceps malalignment was the primary determinant of patellar instability, leading to increased lateralization (bisect offset index up to 0.90, 25% higher than the reference condition), elevated peak contact forces (11% increase), and near-dislocation during knee extension. Valgus alignment increased peak contact forces by up to 3.6% (femur) and 2.9% (tibia) and further exacerbated lateral patellar shift, whereas internal rotation increased peak contact forces by ~ 2.2% and worsened tracking. In contrast, varus alignment and external rotation produced moderate reductions in contact forces (up to − 2.5%) and partial improvement in patellar alignment. The average total computation time was 48 s, and the simulated kinematics and contact forces showed strong agreement with the experimental measurements.

**Conclusion:**

The proposed computational framework enables rapid, subject-specific evaluation of patellofemoral mechanics and implant positioning, incorporating individual anatomical and alignment characteristics. Although demonstrated here in a proof-of-concept setting, its computational efficiency and predictive capability suggest potential for future use in intraoperative assessment and last-minute surgical optimization in TKA.

## Introduction

Total knee arthroplasty (TKA) is widely performed to relieve pain and restore function in patients with advanced knee osteoarthritis or joint degeneration. Despite substantial improvements in implant design and surgical techniques, patellofemoral complications remain a significant clinical concern, occurring in up to 10% of cases and often necessitating revision surgery [1]. The patella plays a critical mechanical role as a sesamoid bone within the quadriceps tendon, acting as a pulley that enhances the quadriceps’ lever arm and contributes to knee extension strength [2]. The patellofemoral joint, formed by the articulation between the patella and the femoral trochlear groove, enables smooth gliding of the patella during flexion and extension. Patellofemoral joint force imbalance can increase contact pressures, cause patellar tilt, subluxation, or dislocation, and negatively affect clinical outcomes [3–5].

Traditional TKA alignment strategies, including kinematic alignment, functional alignment, and gap-balancing, are based on the assumption that the extensor mechanism is oriented perpendicular to either the posterior condylar axis or the axis defined by balanced collateral ligament tension [6–8]. These assumptions inform intraoperative decisions, particularly regarding femoral component rotation and overall limb alignment. However, emerging evidence indicates that additional factors, such as quadriceps malalignment, tibial tubercle position, and soft tissue imbalances, can significantly influence patellar tracking independently of tibiofemoral alignment [9–11]. As a result, restoring the native tibiofemoral joint line or trochlear groove orientation alone may be insufficient to correct pre-existing patellar maltracking in some patients [3].

Over the past several decades, musculoskeletal modeling and simulation have emerged as powerful tools to address these biomechanical challenges [12, 13]. Computational studies have extensively investigated implant positioning, focusing primarily on the alignment of femoral and tibial components [14–17]. These investigations have emphasized joint kinematics, tibiofemoral contact mechanics, and optimization of surgical techniques. Nevertheless, comparatively fewer studies have examined the effects of implant positioning on patellar contact forces and tracking [18–20], leaving an important gap in our understanding of patellofemoral biomechanics following TKA. To address such questions, different computational approaches can be employed, most notably the finite element method (FEM) and multibody dynamics (MBD) [21]. FEM relies on discretizing geometries into meshes of finite elements to capture detailed material behavior and stress–strain distributions, and it has been widely applied to the study of the patellofemoral joint [22–24]. However, the high computational cost associated with mesh generation, simulation, and post-processing limits its applicability in time-constrained clinical settings [25]. As a result, FEM is primarily used for preoperative investigations, such as implant design optimization and surgical planning. In contrast, MBD provides a computationally efficient framework that assumes minimal elastic deformation of bones and implants while accurately capturing joint kinematics and load transmission [18, 26–28]. Recent studies have demonstrated MBD’s utility in simulating knee joint mechanics, including ligament function and contact forces, in near-real time [29]. This efficiency makes MBD particularly suitable for clinical applications, supporting potential use for intraoperative assessment and last-minute surgical optimization by enabling rapid evaluation of surgical strategies and individualized implant positioning, as well as the integration of in vivo measurements acquired during surgery.

In this study, we developed a patient-specific knee model to investigate the effects of quadriceps malalignment on patellar mechanics following total knee arthroplasty. The model included the femur, tibia, and patella, each represented as rigid bodies with implanted components: a femoral component, a tibial baseplate with insert, and a patellar button. These were connected through the main passive stabilizing structures of the knee, namely the medial and lateral collateral ligaments, the patellar tendon, and the medial patellofemoral ligament, while quadriceps loading was applied as the active muscle force driving the system. Bone geometries and tendon attachment sites were reconstructed from medical imaging of a patient with pronounced quadriceps malalignment, while implant geometries were provided by the manufacturer. This formulation provides a subject-specific representation of the patellofemoral joint, capturing both implant articulation and key soft-tissue constraints, thereby enabling reliable prediction of patellar tracking and contact force evolution. A full flexion–extension cycle was simulated to assess patellar tracking and contact forces under different femoral and tibial alignment scenarios, including internal/external rotation and varus/valgus positioning. Both experimental validation using a sensorized 3D-printed knee rig and the computational efficiency of the framework were evaluated to support its potential for practical applications.

## Material and methods

### Quadriceps malalignment and movement analyzed

**Fig. 1:**
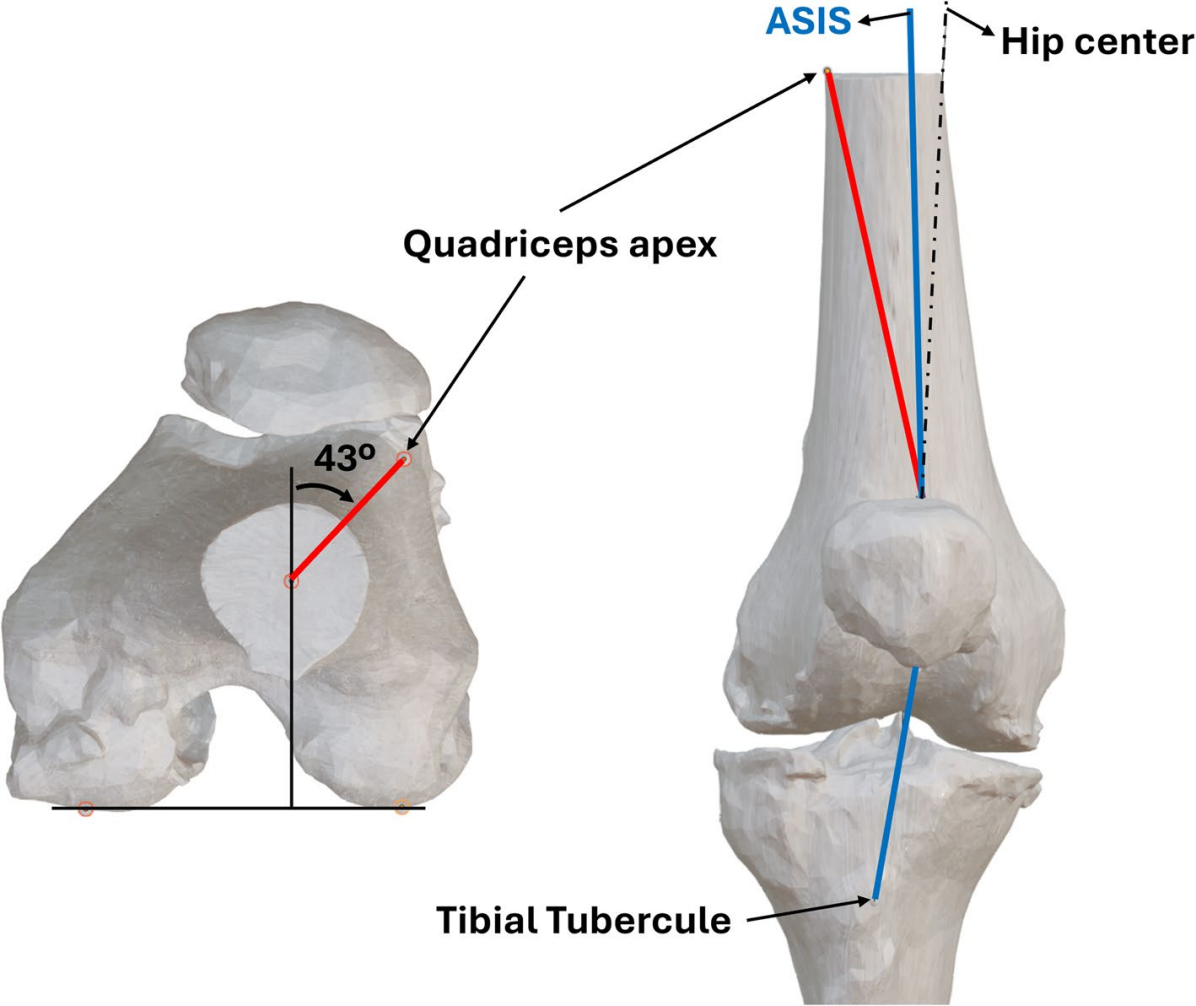
Personalized model of the malalignment case

In this study, quadriceps malalignment is defined by the orientation of the quadriceps tendon relative to the femur (QTA), which differs from the Q-angle, a clinically used measure based on the ASIS that approximates the direction of the quadriceps force acting on the patella. This alignment was quantified using the axial (QTAx) and coronal (QTCA) quadriceps tendon angles, measured from CT imaging; detailed definitions and measurement protocols are provided in [11]. A single representative patient with pronounced quadriceps malalignment was selected for this proof-of-concept study to enable detailed subject-specific modelling and experimental validation under controlled conditions. The subject analyzed was a female patient presenting with pronounced quadriceps malalignment (QTAx: 43.0º; QTCA: 10.3º), a Q-angle of 11.8º, and a hip–knee–ankle (HKA) angle of 1º valgus (Fig. 1). Ethics approval was obtained from the St. Vincent’s Health Network Human Research Ethics Committee (HREC/18/SVH/250), and written informed consent was obtained from the participant for prospective collection and analysis of imaging and outcome data.

To specifically assess the patellofemoral joint, knee extension and flexion were analyzed during active extensor activation while the subject was seated. This motion was chosen because it effectively reveals patterns of patellar maltracking and allows focused evaluation of extensor mechanism alignment [30].

### Computational model

**Fig. 2:**
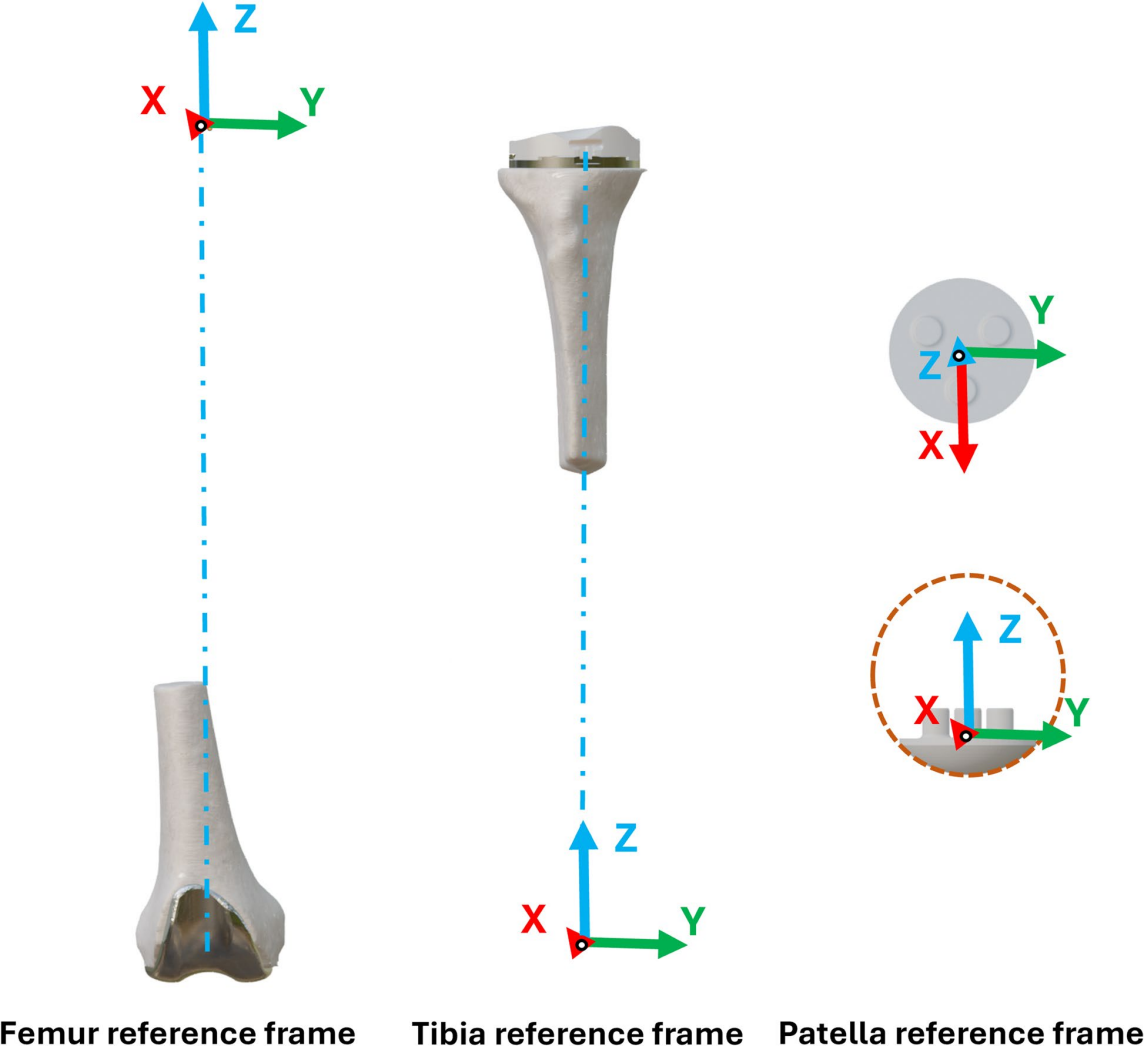
Computational model

The computational leg model in this study consisted of three rigid bodies, each incorporating its respective implant: the femur, the tibia-foot assembly, and the patella (Fig. 2). Three-dimensional bone geometries were reconstructed from CT imaging data in DICOM format through segmentation using Materialise Mimics (Materialise, Leuven, Belgium). The segmentation process, performed by a trained medical imaging professional, combined intensity thresholding, region growing, and manual refinement to ensure accurate representation of the cortical bone surfaces. Anatomical landmarks and soft tissue insertion points were subsequently identified on the reconstructed geometries based on established anatomical references and morphological features. Implant geometries, including the femoral component, tibial baseplate with insert, and patellar button, were provided by the manufacturer and integrated into the model to reproduce the postoperative configuration.

During the simulation, the femur was fixed in a horizontal position, while the tibia and patella were modeled as free bodies, each with six degrees of freedom. The tibia and femur were connected via the collateral ligaments, the tibia and patella were linked through the patellar tendon, modeled as two discrete tendons, and the femur and patella were connected via the medial patellofemoral ligament (MPFL); all tendons and ligaments were modeled as linear extension spring-damper elements [31], active only in tension and characterized by constant stiffness. Quadriceps contraction was modeled using an actuator that applied a tensile force between the patella and femur. This actuator drove a controlled knee extension-flexion cycle, moving the joint from 80° to 10° of flexion at a constant prescribed velocity.

Contact interactions were modeled between the femoral implant and tibial insert, between the femoral and patellar implants, and for the wrapping of the medial collateral ligament (MCL) around the tibia and the MPFL around the femur.

The subject-specific parameters, together with the mechanical constraints of the system, were incorporated into a custom-developed modeling framework [32]. The body-fixed reference frames assigned to the rigid segments are shown in Fig. 2. For the femur, the reference frame was defined using the mechanical axis extending from the center of the femoral head to the center of the knee joint, along with the medial-lateral axis passing through the medial and lateral epicondyles (Y-axis). The patellar reference frame was established based on its longitudinal axis (X-axis), a medial-lateral axis aligned with that of the femur (Y-axis), and an anterior-posterior axis (Z-axis) orthogonal to the other two directions [33].

### Treatment strategies and reference

To evaluate the influence of different surgical strategies on patellofemoral mechanics, several implant positioning configurations were simulated.

i) Standard treatment: The femoral and tibial components were positioned by an experienced orthopedic surgeon based on Kinematic Alignment theory to recreate the patient’s native anatomy and bone morphology, without performing ligament balancing (which would require additional patient-specific data). The positioning was chosen to minimize collateral ligament tension in order to specifically investigate the influence of implant positioning on the extensor mechanism. The hip–knee–ankle (HKA) alignment was preserved by applying neither varus nor valgus correction, and the femoral component rotation reproduced the orientation of the native posterior condyles.
ii) Femoral varus and valgus: Based on the standard configuration, a varus and a valgus correction of 3° was applied to the femoral component only, simulating common coronal alignment adjustments during surgery.
iii) Tibial varus and valgus: Based on the standard configuration, a varus and a valgus correction of 3° was applied to the tibial component only, representing typical tibial alignment variations.
iv) Femoral internal and external rotation: Based on the standard configuration, an internal and an external rotation of 4° were applied to the femoral component only, reflecting clinically relevant rotational adjustments.

To further investigate the role of quadriceps alignment in patellofemoral behavior, three different quadriceps force directions were considered:

i) QTAx 43°: This direction corresponds to the native position of the quadriceps apex in the malaligned patient and represents the pathological preoperative condition. It was applied in all treatment configurations.
ii) QTAx 0°: This direction represents a corrected quadriceps alignment corresponding to an idealized postoperative configuration. It was introduced as a reference to quantify the potential improvement achievable through implant positioning. This condition was applied only in the standard treatment configuration to maintain a consistent reference.
iii) ASIS: This direction corresponds to the conventional clinical definition of the quadriceps force line based on the anterior superior iliac spine (ASIS), commonly used to define the Q-angle and extensor mechanism alignment [34]. It was applied only in the standard treatment configuration to illustrate the clinical implications of different definitions of quadriceps force orientation

### Simulation

#### Formulation

In this study, the dynamics of the multibody system were formulated using the ALI3-P approach. This formulation, originally introduced in [35], has been progressively refined over time and is grounded in earlier developments reported in [24] and [25]. ALI3-P is an Augmented Lagrangian-based method, specifically an index-3 formulation expressed in mixed coordinates that combine natural and relative coordinate representations. The formulation accounts for constraint enforcement through velocity and acceleration projections onto the corresponding constraint subspaces. A detailed presentation of the governing equations of motion, as well as the velocity and acceleration projection procedures, can be found in [36].

Time integration of the system equations was performed using the Newmark integration scheme [37], employing a fixed time step of 1 ms.

#### Static equilibrium

The tibia and patella were initially placed near their expected static equilibrium configuration; however, this equilibrium state had to be determined explicitly. For dynamic simulation of a multibody system, it is essential to define initial positions and velocities that satisfy the constraint equations at both the position and velocity levels. When a system exhibits a static equilibrium configuration, initializing the simulation from this state is preferable, as it avoids large initial accelerations that could compromise numerical stability.

Determining such an initial condition requires solving the static equilibrium equations of the system. However, when contact interactions between bodies are present, the static equilibrium problem becomes considerably more complex, and in some cases may admit multiple solutions, making its resolution challenging. The resulting nonlinear system was solved using a Newton–Raphson iterative scheme, consistent with the approach employed to solve the equations of motion [36].

#### Contact model

Because the contacting surfaces are lubricated by synovial fluid and the experimental contact interface was similarly lubricated, contact interactions were modeled using only normal contact forces, while tangential (frictional) effects were neglected. The normal contact force was described using the Flores contact model [38], expressed as:

1 $${\mathbf F}_{\mathrm n}={\mathrm k}_{\mathrm n}\delta^p\left(1+\frac{8\left(1-\varepsilon\right)}{5\varepsilon}\frac{\dot\delta}{{\dot\delta}_0}\right)\mathbf n,$$

where ${k}_{n}$ represents the equivalent contact stiffness, which is determined by the geometry and material properties of the contacting bodies; *p* is Hertz’s exponent; $\delta$ denotes the indentation and $\dot{\delta }$ its temporal derivative; ${\dot{\delta }}_{0}$ is the relative normal velocity between the bodies at the moment contact is detected; is the coefficient of restitution $\varepsilon$; and **n** is the direction of the force. The subscript “n” refers to the normal component.

#### Contact detection

**Fig. 3:**
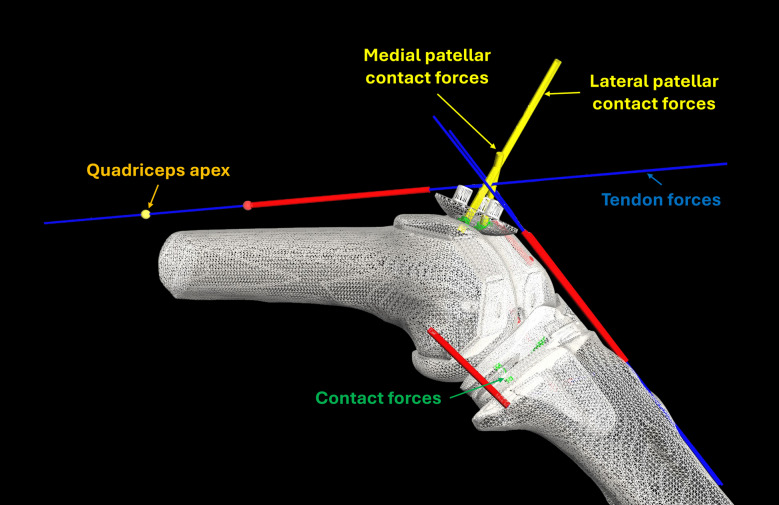
Simulation of contact and tendon forces

A key computational difficulty in the integration of contact phenomena within multibody dynamics concerns the reliable detection of collisions. The development of numerical strategies capable of reproducing contact interactions with both accuracy and computational efficiency, while ensuring physically realistic behavior of the multibody system, remains a demanding task. This issue becomes particularly critical when the interacting bodies exhibit complex three-dimensional geometries, for which advanced and robust detection schemes are required. In the present work, the intricate three-dimensional geometries of the bodies were represented by surface discretizations composed of triangular elements. During knee extension, contact occurred between the femoral and tibial implants, for which a mesh-to-mesh contact detection strategy was applied. In contrast, the patellar implant, exhibiting an approximately spherical geometry, was modeled as a primitive shape, allowing the use of a more computationally efficient detection scheme to identify interactions with the femoral component. Specifically, a sphere-to-mesh detection algorithm [39] was employed, which determines contact by evaluating the minimum distance between the sphere surface and the surrounding mesh (Fig. 3). The radius and center of the spherical representation were defined to fit the inferior articular surface of the commercial patellar implant used in this study, ensuring an accurate approximation of the functional contact region while maintaining computational efficiency. This approach provides an effective and accurate means of detecting contact between a spherical body and complex three-dimensional surfaces [29]. This efficient contact detection technique was also applied to simulate the wrapping of muscles and tendons around the bones. The two contact detection methodologies were implemented in the in-house library [32].

The triangular meshes of the contacting components (femoral implant and tibial insert) were generated from the native CAD geometries using Gmsh, with a maximum element size of 2.5 mm.

The algorithm identifies all contact regions between the spherical implant and the mesh, corresponding to disconnected contact zones, and computes a normal force for each region, typically resulting in multiple local contact forces. To estimate the total contact force acting on the patella, the resultant of all detected forces and the corresponding point of application were calculated. In addition, to characterize mediolateral imbalances in patellar contact loading induced by quadriceps malalignment [11], the resultant forces on the lateral and medial sides were evaluated separately. This was achieved by summing the contact forces whose points of application were located laterally or medially with respect to the patellar XZ reference plane (Fig. 3 in yellow).

### Experimental data collection and experimental validation

**Fig. 4:**
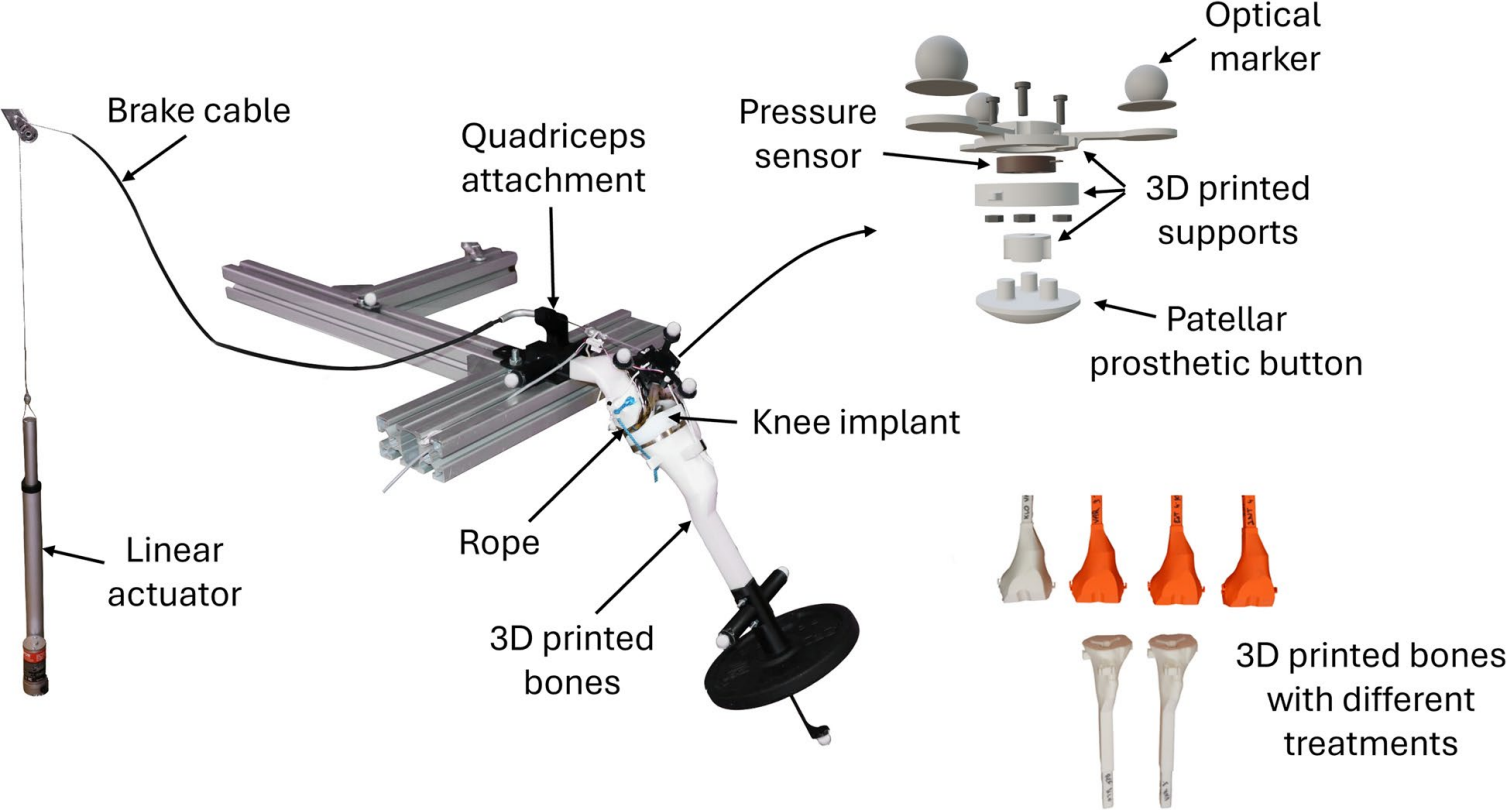
Modified sensorized knee test rig

To verify the accuracy of the simulations in reproducing patellofemoral mechanics, experimental validation was conducted using a sensorized 3D-printed knee rig (Fig. 4), in which patellar normal contact forces and motion were directly measured [40]. Although cadaveric experiments could provide greater anatomical realism, they involve higher costs and ethical constraints. Moreover, cadaveric specimens do not permit systematic comparisons of multiple treatment strategies within the same subject, as bone resections can only be performed once. In contrast, 3D-printing technology enables precise reproduction of planned treatments and facilitates controlled, repeatable comparisons across different configurations.

The bones were virtually segmented, digitally cut, and subsequently 3D-printed using an X1 Carbon printer (Bambu Lab, Shenzhen, China) to allow implantation of commercial tibial and femoral components (MicroPort®). The patellar prosthesis was mounted on a sensorized 3D-printed base, which differed slightly from the patient-specific bone geometry, being larger and wider. Tendons were replicated using ropes. The femur was rigidly fixed, and each trial began with the knee positioned at 80° of flexion. A complete extension-flexion cycle was then generated by a linear actuator simulating quadriceps contraction at a constant speed.

Kinematics of the femur, tibia, and patella were recorded from the trajectories of eight optical markers tracked by 18 infrared cameras (OptiTrack FLEX 3, Natural Point, Corvallis, OR, USA) at a sampling frequency of 100 Hz. Simultaneously, the contact force between the femur and the patellar prosthetic button was measured using a compact pressure load cell (FX29, TE Connectivity, Wört, Germany), also sampled at 100 Hz. Marker trajectories were filtered using a second-order Butterworth filter with a 12 Hz cutoff frequency [41], while force signals were smoothed using singular spectrum analysis (SSA) [42] with a window length of 20.

The positions and orientations of the rigid bodies were determined from the marker data using the conventional approach described by Vaughan [43] which involves: (i) selecting three non-collinear entities (markers or predefined joint locations) for each segment; (ii) defining an orthogonal reference frame for each segment based on these entities; and (iii) applying correlation equations to compute the rigid body position and orientation.

All configurations were experimentally evaluated by interchanging the femoral and tibial bones according to the applied treatment. Actuation of the linear actuator was performed from two different positions corresponding to the malaligned and corrected configurations, enabled by a dedicated 3D-printed base (Fig. 4). The ASIS-based force direction was not experimentally tested, as it would have required substantial modification of the experimental setup.

The experimental measurements allow for comparison with the simulation of the following variables:

- Patellar trajectory relative to the femoral implant
- Patellar rotation, with a particular focus on tilt
- Patellar contact forces

As the patellar pressure sensor measured only vertical forces, only the force component along the patellar local Z-axis (Fig. 2) was considered for comparison with the simulation results. It is also important to note that the numerical simulations were performed independently of the experimental data, with no experimental measurements used as input parameters, thereby enabling an unbiased validation of the model predictions. Given the controlled experimental conditions ensured by the use of a linear actuator, which minimized operator-dependent variability and provided highly repeatable motion, a single representative experimental trial per configuration was considered sufficient, as consistent behavior was observed across preliminary repetitions.

### Computational details

The calculations were conducted on a computer equipped with an Intel(R) Core(TM) i7-13700KF @ 3.40 GHz processor, 32 GB RAM, and a 2 TB SSD running Windows 10 Pro. The analysis was performed using a single-threaded program written in Fortran 2008 and C + +. The program was compiled using MSVC 2017 and Intel Fortran 2018. Efficiency was gauged by measuring runtime, distinguishing the time needed for obtaining the initial static equilibrium configuration and for executing the simulation. The algorithm is inherently sequential, as most computational steps depend on the results of the immediately preceding step, which limits straightforward parallelization. Nevertheless, several optimizations were implemented at the timestep level to reduce computational cost, such as reusing the list of potentially colliding surfaces across successive iterations or timesteps.

## Results

This section presents the numerical simulation results for the different configurations described previously, together with their corresponding experimental validation.

### Quadriceps force vector direction effects

#### Patellar kinematics

**Fig. 5:**
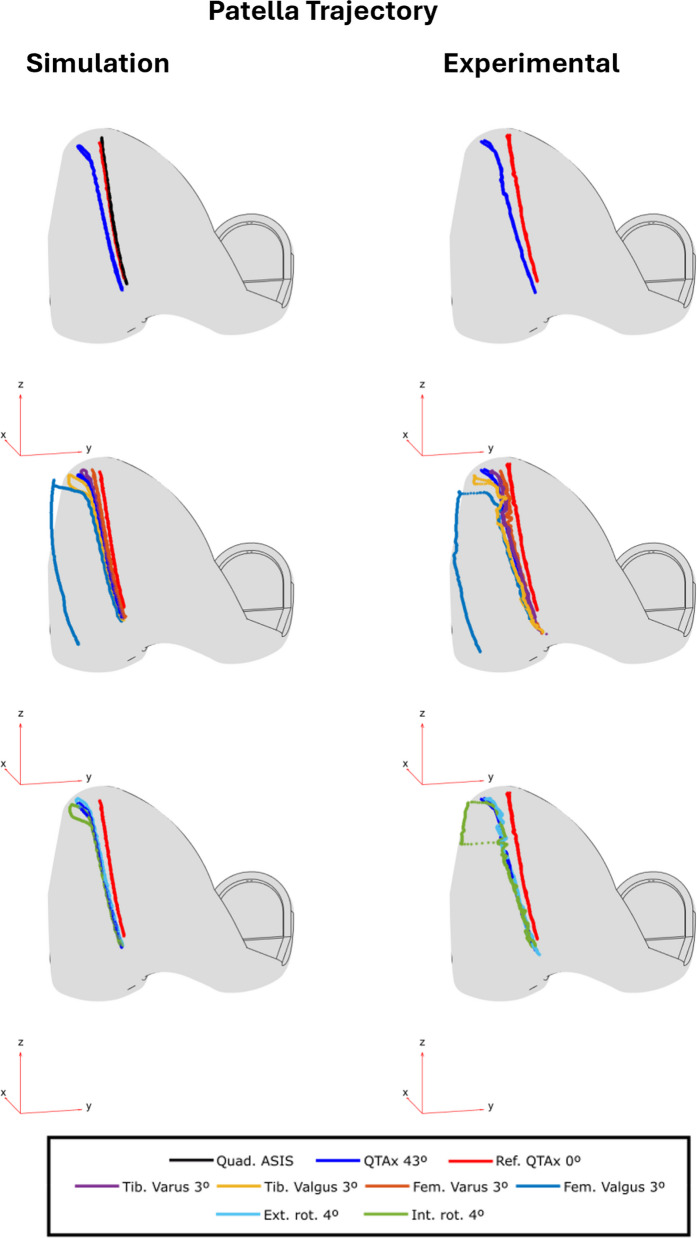
Patella trajectory

**Fig. 6:**
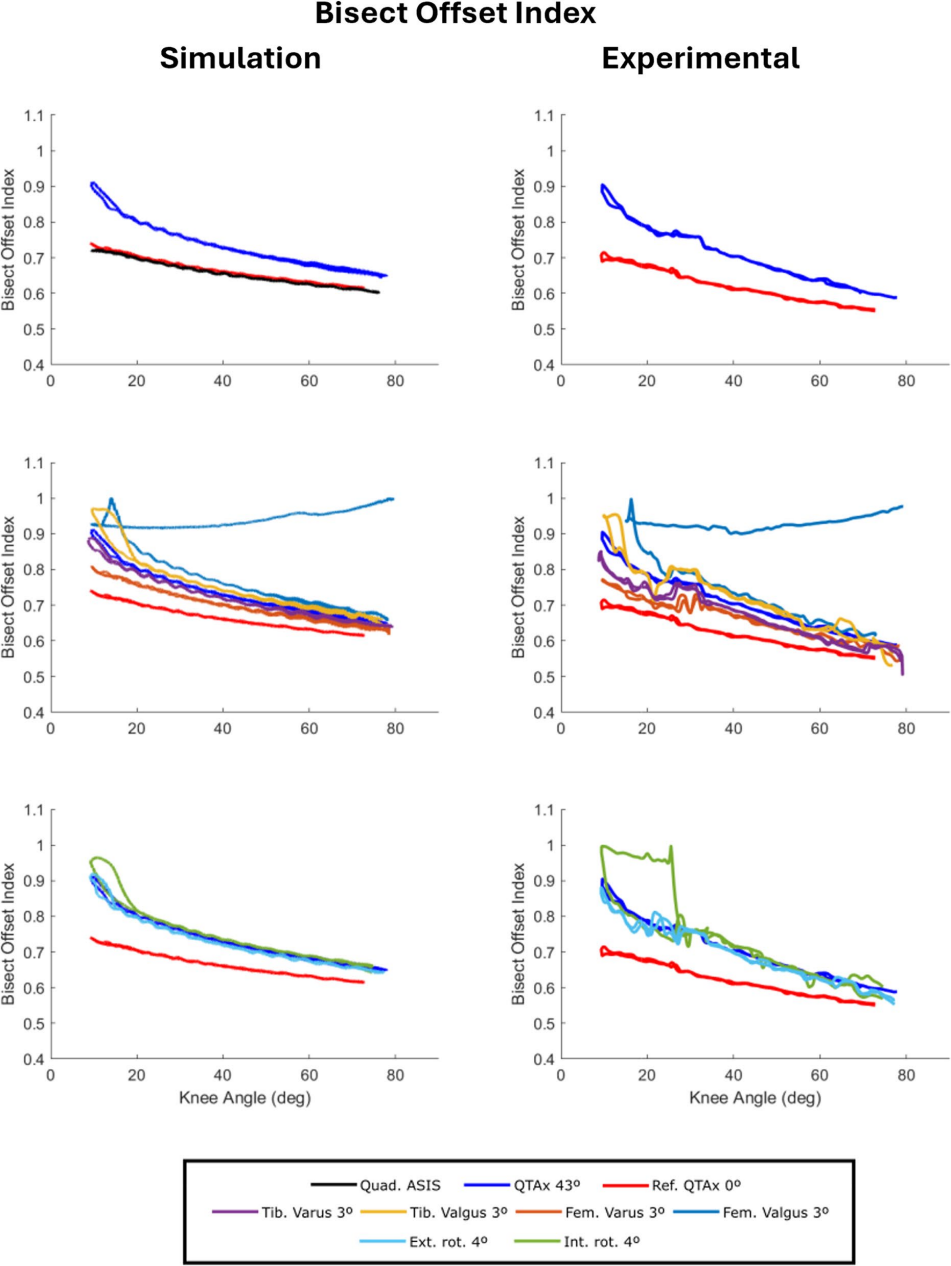
Bisect Offset Index

Figure 5 shows the patellar trajectories on the femoral implant obtained from numerical simulations (left column) and experimental measurements (right column) for the different quadriceps force directions (first row). The corresponding evolution of the bisect offset index [44] is presented in Fig. 6.

When the patient-specific malaligned quadriceps apex was used to define the force direction (blue), the patellar trajectory exhibited pronounced lateralization compared with the well-aligned reference condition (red). The bisect offset index increased up to 0.9, resulting in a near-dislocation at approximately 15° of knee extension. In contrast, when the quadriceps force direction was defined using the ASIS (black), the resulting trajectory closely matched the reference configuration, with a maximum bisect offset index below 0.75.

**Fig. 7:**
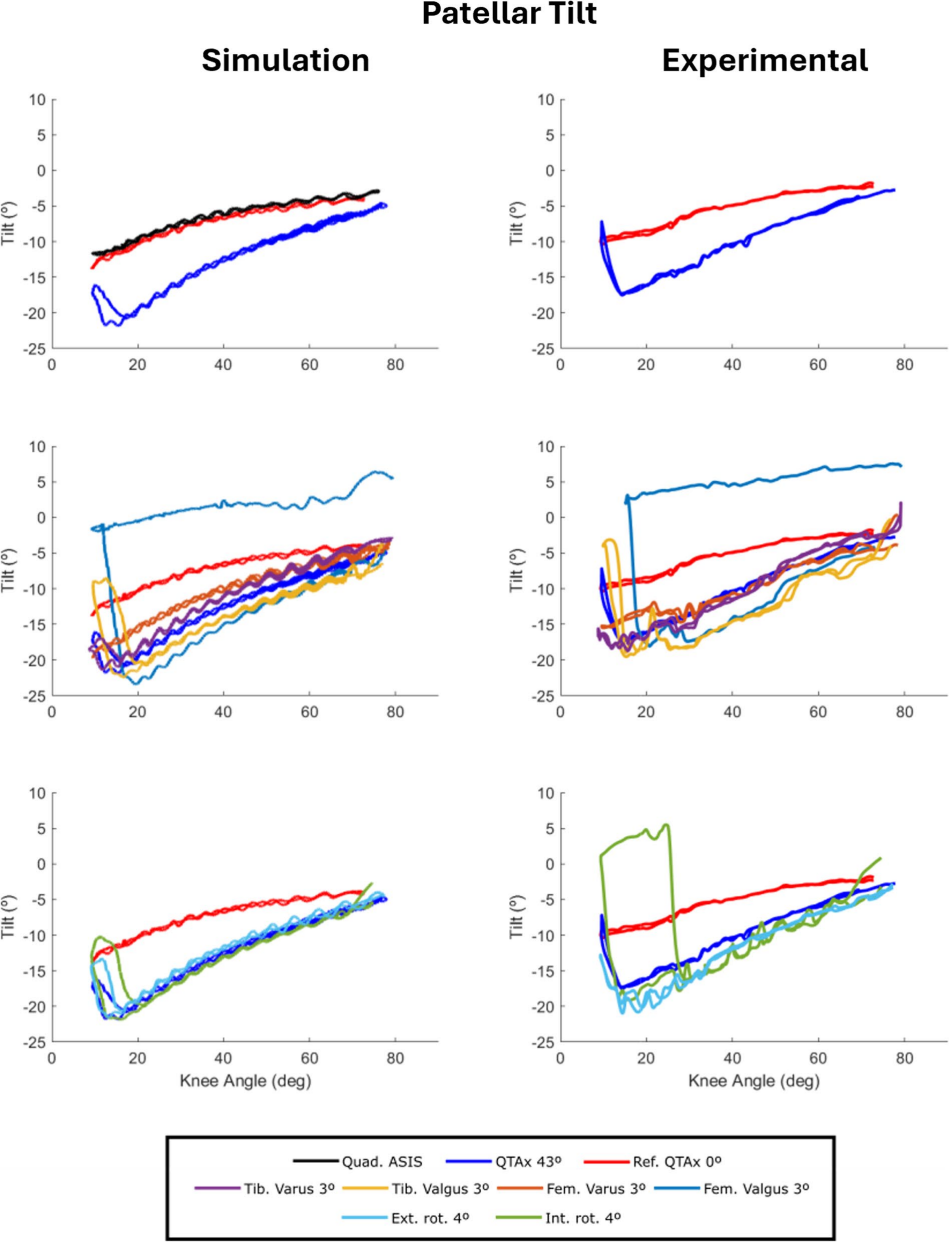
Patellar tilt

Comparable trends were observed for patellar tilt (Fig. 7). Quadriceps malalignment led to increased tilt, and the near-dislocation observed at approximately 15° of extension was also evident in the tilt measurements.

#### Contact forces

**Fig. 8:**
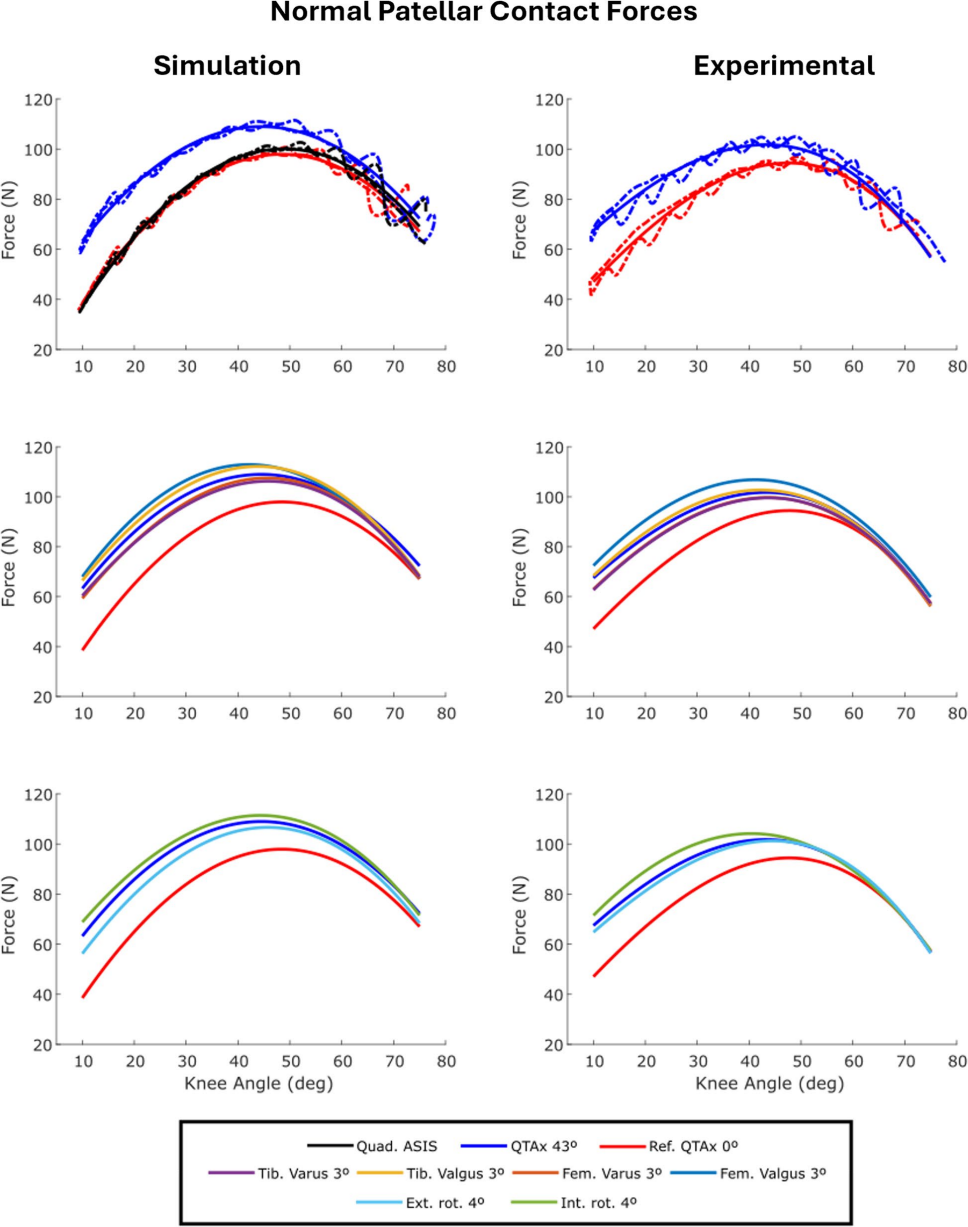
Normal patellar contact forces

Differences in patellar contact forces between quadriceps force directions were greater in knee extension than in knee flexion (Fig. 8). Raw data from numerical simulations (left column) and experimental measurements (right column) are displayed using dashed lines. Oscillations were present in both datasets, primarily due to stick-slip effects at the contact interface.

To facilitate comparison across the various configurations, the force-flexion angle relationships were approximated using third-order polynomial fits (solid lines). Up to 60° of knee flexion, contact forces were comparable among all conditions. However, below 30°, substantial differences emerged, with significantly higher forces in the malaligned quadriceps configuration (blue). Peak contact forces occurred at approximately 45° of flexion, reaching values close to 100 N in the malaligned case, 11% higher than the reference case (red).

**Fig. 9:**
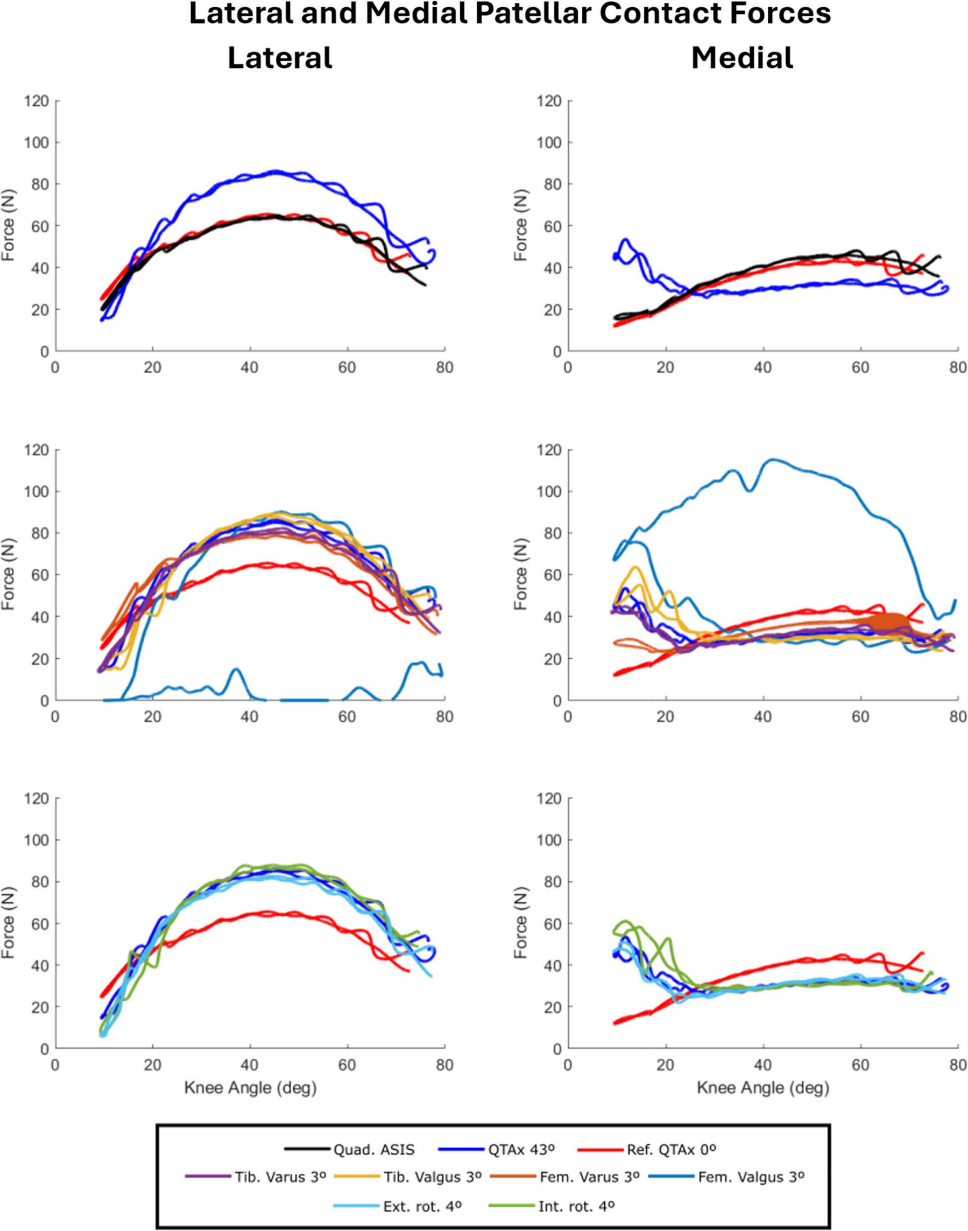
Lateral and medial patellar contact forces

The mediolateral distribution of contact forces further illustrates the imbalance induced by quadriceps malalignment (Fig. 9). Under this condition, lateral contact forces were approximately 2.5 times greater than medial forces (blue). Consistent with the kinematic results, the ASIS-based force direction (black) produced force distributions similar to those observed in the well-aligned reference condition (red).

### Varus/valgus effects

#### Patellar kinematics

The effects of introducing varus and valgus alignment at the tibial or femoral level on patellar trajectories are presented in Fig. 5 (second row). The corresponding evolution of the bisect offset index is shown in Fig. 6.

Applying 3° of valgus alignment to either the tibial or femoral component substantially degraded patellar tracking, increasing lateralization and the bisect offset index (reaching maxima of 0.96 and 0.99, respectively). In the case of tibial valgus, the patella exhibited a brief dislocation during knee extension but was able to re-enter the trochlear groove during flexion (yellow). In contrast, femoral valgus resulted in a persistent dislocation, with the patella completing the flexion phase outside the trochlear groove (steel blue). This behavior was consistently observed in both the numerical simulations and the experimental measurements (Fig. 5).

Conversely, applying 3° of varus alignment to either component significantly improved patellar tracking by promoting medialization and reducing the bisect offset index to 0.89 and 0.81 for tibial and femoral varus, respectively. Femoral varus (orange) produced greater improvement, approaching the reference condition (red), whereas tibial varus (purple) yielded more moderate benefits.

Similar patterns were observed for patellar tilt (Fig. 7). Valgus alignment increased tilt (up to 22° and 24° for tibial and femoral valgus, respectively), with dislocations near full extension also reflected in the tilt measurements. In contrast, femoral varus alignment reduced tilt to 19.5°.

#### Contact forces

At the force level, trends consistent with the kinematic results were observed (Fig. 8). Femoral valgus notably worsened mediolateral force balance (Fig. 9) and increased the maximum normal contact force by 3.6%, while tibial valgus produced a 2.9% increase. In contrast, femoral and tibial varus alignment led to slight reductions in peak contact forces, by 1.4% and 2.5%, respectively.

Similar trends were identified in the experimental measurements (Fig. 8, right column). Despite the improvements observed under varus alignment, contact force levels remained substantially higher (106 N) than those of the reference condition with optimal quadriceps alignment (98 N). Comparable observations were made for mediolateral force distribution (Fig. 9): although varus alignment partially restored balance, it did not achieve the symmetry observed in the reference configuration. Persistent dislocation under femoral valgus alignment was again evident in the force measurements (steel blue).

### Internal/external rotation effects

#### Patellar kinematics

The influence of femoral internal and external rotation on patellar tracking is illustrated in Fig. 5 (third row). The corresponding evolution of the bisect offset index is presented in Fig. 6.

External rotation of the femoral component had minimal influence on patellar kinematics. The original treatment configuration (blue) and the externally rotated condition (cyan) exhibited very similar trajectories, bisect offset index values (maxima of 0.91 and 0.92), and patellar tilt (maxima of 21.7° and 21.6°), indicating nearly identical tracking behavior throughout the flexion-extension cycle.

In contrast, the introduction of internal rotation resulted in a slight deterioration of patellar tracking. Increased lateralization was observed, accompanied by higher bisect offset index values (maximum of 0.96) and a brief episode of patellar subluxation during knee extension. These trends were consistently observed in both numerical simulations and experimental measurements (Fig. 5).

Similar effects were observed for patellar tilt (Fig. 7). External rotation did not substantially improve tilt, whereas internal rotation led to increased tilt and reduced patellar stability.

#### Contact forces

Consistent with the kinematic findings, femoral rotation also influenced patellofemoral contact forces (Fig. 8, third row). External rotation reduced the peak contact force by 2.2%, whereas internal rotation increased it by 2.2%. Similar trends were observed in the experimental measurements (Fig. 8, right column).

A comparable pattern was identified in the mediolateral distribution of contact forces (Fig. 9). External rotation slightly improved load balance between the medial and lateral compartments, while internal rotation further aggravated lateral force dominance.

### Experimental validation

A quantitative comparison between numerical simulations and experimental measurements is summarized in Table 1. This table reports the root mean square error (RMSE) of the contact forces, the differences between simulated and experimental peak contact forces, as well as the discrepancies in the bisect offset index and patellar tilt for all investigated configurations. These objective metrics provide a comprehensive assessment of the accuracy of the proposed model in reproducing both patellofemoral kinematics and contact loading under different treatment conditions.

**Table 1:** Comparison between simulation and experimental measurements

		**Ref. QTAx 0º**	**QTAx 43º**	**Fem. Valgus 3º**	**Fem. Varus 3º**	**Tib. Valgus 3º**	**Tib. Varus 3º**	**Int. rot. 4º**	**Ext. rot. 4º**	**Mean**
RMSE	Contact forces (N)	8.5	4.2	4.5	3.7	1.9	2.3	2.6	8.6	**4.5**
Dif. between maximum values	Contact forces (N)	3.5	7.2	6.1	7.8	9.5	6.7	7.3	5.4	**6.7**
	Bisect Offset Index	0.03	−0.01	0.00	0.04	0.02	0.04	−0.03	0.03	**0.01**
	Patellar tilt (º)	−2.1	−1.7	−1.1	−3.4	−2.9	−4.8	−8.1	−1.1	**−2.8**

Overall, good agreement was observed between numerical and experimental results across all configurations, confirming the ability of the model to reliably reproduce patellofemoral mechanics. For contact forces, the RMSE remained limited, with a mean value of 4.5 N. The lowest discrepancies were observed for the tibial varus and valgus configurations, whereas higher errors were recorded for the reference (QTAx 0°) and internal rotation cases. Differences between simulated and experimental peak contact forces were also moderate, with a mean absolute difference of 6.7 N, indicating that the model accurately captured both the magnitude and temporal evolution of patellofemoral contact forces.

Regarding patellar tracking, the bisect offset index showed excellent agreement between simulations and experiments, with a mean difference of 0.01. Across all configurations, discrepancies remained below 0.04, demonstrating that the model reliably reproduced mediolateral patellar positioning.

Similarly, patellar tilt was predicted with reasonable accuracy. The mean difference between simulated and experimental tilt values was − 2.8°, suggesting a slight systematic underestimation in the numerical model. Larger discrepancies were observed in the internal rotation configuration (− 8.1°), which can be attributed to a more pronounced patellar dislocation in the experimental setup.

These quantitative results are consistent with the qualitative observations presented in the previous sections, where similar trends in patellar tracking, stability, and contact loading were identified in both experimental and numerical analyses. In particular, the model successfully reproduced the deteriorating effects of valgus alignment and internal rotation, as well as the stabilizing influence of varus alignment and optimized quadriceps orientation.

### Computational efficiency

The average time for calculating the initial position was 14 s, the average time for simulating extension and flexion was 34 s, and the average total computation time was 48 s.

## Discussion

This study evaluated the influence of quadriceps force direction, varus/valgus alignment, and femoral rotation on patellofemoral kinematics and contact forces, combining numerical simulations with experimental validation. The results demonstrate the critical role of quadriceps alignment and implant positioning in maintaining patellar stability and balanced contact loading.

### Quadriceps force direction

Both simulations and experiments consistently showed that a malaligned quadriceps apex substantially increases lateralization of the patella, resulting in higher bisect offset index values, increased patellar tilt, and elevated lateral contact forces. In extreme cases, a near-dislocation occurred during knee extension. In contrast, defining the quadriceps force direction using the ASIS or a well-aligned reference configuration restored patellar tracking and balanced mediolateral load distribution. These findings are consistent with prior cadaveric studies demonstrating the influence of quadriceps force direction on patellofemoral kinematics [45]. Malalignment of the quadriceps force shifts contact forces laterally, creating mediolateral imbalance that may contribute to lateral facet patellofemoral osteoarthritis, as recently reported in subjects with quadriceps tendon malalignment [11]. The pronounced differences observed when comparing force directions toward the ASIS versus the quadriceps apex highlight the critical importance of this variable, warranting further investigation. These findings may have implications for redefining extensor mechanism alignment and the clinical measurement of the Q-angle [34].

### Varus/valgus alignment

Small angular deviations at the femoral or tibial component had a pronounced effect on patellar tracking and loading. Valgus alignment, particularly at the femur, worsened lateralization and could induce persistent patellar dislocation, whereas varus alignment improved medialization and partially restored normal tracking. These results are consistent with previous studies demonstrating that valgus malalignment increases Q-angle by lateralizing the tibial tubercle [46]. These observations closely align with the recent findings of Chesney et al. [20], who reported that valgus alignment increases patellar spin, tilt, and lateral shift, whereas varus alignment reduces these parameters.

Although varus alignment improved patellar mechanics, contact forces and mediolateral balance remained inferior to those observed in the reference configuration with optimized quadriceps alignment. This indicates that coronal correction alone is insufficient to fully compensate for extensor mechanism malalignment and highlights the multifactorial nature of patellofemoral stability. Altering coronal alignment away from a patient’s constitutional alignment is also likely to cause significant collateral ligament imbalance.

### Internal and external femoral rotation

Axial alignment of the femoral component also influenced patellofemoral mechanics. External rotation had minimal impact on patellar tracking and contact forces, producing results similar to the original treatment configuration. Conversely, internal rotation moderately worsened patellar stability, increased lateralization, and elevated contact forces, occasionally resulting in transient subluxation. Although only limited differences were observed in this case study, these trends are consistent with the conclusions found in the literature [20, 47, 48], which indicate that internal rotation increases patellar tilt and lateral shift, whereas external rotation reduces these parameters.

In addition, a recent retrospective analysis of prospectively collected data has also reported that outcomes in patients with quadriceps malalignment can be improved by externally rotating the femoral component to accommodate extensor mechanism deformity [3]. The present study confirms that internal rotation exacerbates lateral patellofemoral loading, while external rotation provides stabilization.

### Experimental validation

Quantitative comparison with experimental measurements demonstrated good agreement across all configurations. RMSE values for contact forces were low (mean 4.5 N), bisect offset index differences were minimal (mean 0.01), and patellar tilt predictions were within 2–3° on average. These results confirm the reliability of the numerical model in reproducing patellofemoral kinematics and contact loading under a range of clinically relevant implant positions.

### Computational efficiency

Beyond biomechanical accuracy, the proposed modeling framework demonstrated sufficient computational efficiency to support time-constrained clinical applications. The adopted contact detection and force formulation enabled stable simulations of full flexion-extension cycles within practical computation times, despite the complexity of multibody dynamics and contact interactions. This level of performance is particularly relevant for intraoperative use, where rapid simulations could support real-time assessment of implant positioning, direct intraoperative measurements, and last-minute preoperative or surgical adjustments. By enabling near real-time feedback on patellofemoral mechanics, the proposed approach has the potential to assist surgeons in optimizing alignment decisions directly during surgery.

### Limitations

Despite the strong agreement with experimental data, this study has several limitations. In the experimental setup, tendons were represented using synthetic ropes rather than physiological or commercially validated ligament substitutes, which may introduce deviations in absolute force transmission; however, this approach was chosen to ensure repeatability and avoid the ethical and practical limitations associated with biological tissues. The model relies on a simplified anatomical representation to maintain computational efficiency and to avoid ethical and economic constraints associated with experimental validation. As a result, it may not fully capture the complex geometry, soft tissue heterogeneity, or viscoelastic behavior of the human knee. Future work will aim to increase anatomical realism by incorporating the lateral patellofemoral ligament and modeling the quadriceps as multiple force lines, while preserving manageable simulation times.

Ligament contributions were also deliberately minimized to reduce the number of variables and avoid confounding effects. Consequently, ligament tension was not actively adjusted to reflect surgical balancing procedures, and the reported contact forces primarily reflect the influence of implant positioning and quadriceps force direction under controlled conditions. In a clinical setting, ligament balancing would significantly influence joint loading and may alter the absolute magnitude of the predicted forces. Therefore, the contact force results should be interpreted as comparative indicators between configurations rather than direct physiological predictions. Furthermore, the results presented here are specific to the analyzed subject and are not intended to support generalized conclusions, as different patients may exhibit different biomechanical responses depending on their anatomical and soft tissue characteristics. Nonetheless, preliminary observations suggest that the developed framework is capable of incorporating ligament balancing effects, as increases in ligament stiffness were associated with elevated contact forces, supporting its potential for more comprehensive modeling in future developments.

Finally, the study focused on isolated knee extension under controlled conditions, which may not reflect the full range of dynamic activities encountered in daily life. Furthermore, only a single patient was analyzed, limiting the generalizability of the results. This also constrained the exploration of patient-specific synergistic effects between quadriceps force direction and implant positioning, which would require additional cases with different quadriceps alignment patterns and is left for future work. Nevertheless, similar trends were reported by Chesney et al. [20] in simulations of weight-bearing knee bending across 10 subjects. Importantly, the model is intended to support relative comparisons between surgical scenarios at the subject-specific level, rather than to provide absolute force measurements for direct clinical decision thresholds. Within this intended use, the observed level of accuracy is considered sufficient to discriminate between treatment options and guide surgical interpretation.

## Conclusions

This study suggests that patient-specific multibody dynamics simulations can reproduce patellar tracking and contact force trends following total knee arthroplasty, offering a useful framework to explore the biomechanical influence of quadriceps malalignment and implant positioning. In the investigated subject-specific case, quadriceps malalignment appeared to be a key contributor to lateral patellar displacement, increased tilt, and elevated contact forces, while valgus alignment and internal rotation tended to exacerbate these effects. In contrast, varus alignment and external rotation showed moderate improvements in patellofemoral mechanics. The simulations were supported by experimental validation and enabled rapid evaluation of multiple alignment scenarios, highlighting their potential for subject-specific preoperative planning and intraoperative decision support. The proposed computational framework enables rapid, subject-specific evaluation of patellofemoral mechanics and implant positioning, incorporating individual anatomical and alignment characteristics. Although demonstrated here in a proof-of-concept setting, its computational efficiency and predictive capability suggest potential for future use in intraoperative assessment and last-minute surgical optimization in TKA.

## Data Availability

No datasets were generated or analysed during the current study.
